# Divergent evolution of terrestrial locomotor abilities in extant Crocodylia

**DOI:** 10.1038/s41598-019-55768-6

**Published:** 2019-12-17

**Authors:** John R. Hutchinson, Dean Felkler, Kati Houston, Yu-Mei Chang, John Brueggen, David Kledzik, Kent A. Vliet

**Affiliations:** 1 Structure and Motion Laboratory, Department of Comparative Biomedical Sciences, The Royal Veterinary College, Hawkshead Lane, North Mymms, AL9 7TA United Kingdom; 2 St Augustine Alligator Farm and Zoological Park, St Augustine, Florida USA; 3 Research Support Office, The Royal Veterinary College, Royal College Street, London, NW1 0TU United Kingdom; 4 University of Florida, Department of Biology, 208 Carr Hall, PO Box 118525, Gainesville, Florida 32611-8525 USA

**Keywords:** Evolution, Biomechanics, Herpetology

## Abstract

Extant Crocodylia are exceptional because they employ almost the full range of quadrupedal footfall patterns (“gaits”) used by mammals; including asymmetrical gaits such as galloping and bounding. Perhaps this capacity evolved in stem Crocodylomorpha, during the Triassic when taxa were smaller, terrestrial, and long-legged. However, confusion about which Crocodylia use asymmetrical gaits and why persists, impeding reconstructions of locomotor evolution. Our experimental gait analysis of locomotor kinematics across 42 individuals from 15 species of Crocodylia obtained 184 data points for a wide velocity range (0.15–4.35 ms^−1^). Our results suggest either that asymmetrical gaits are ancestral for Crocodylia and lost in the alligator lineage, or that asymmetrical gaits evolved within Crocodylia at the base of the crocodile line. Regardless, we recorded usage of asymmetrical gaits in 7 species of Crocodyloidea (crocodiles); including novel documentation of these behaviours in 5 species (3 critically endangered). Larger Crocodylia use relatively less extreme gait kinematics consistent with steeply decreasing athletic ability with size. We found differences between asymmetrical and symmetrical gaits in Crocodylia: asymmetrical gaits involved greater size-normalized stride frequencies and smaller duty factors (relative ground contact times), consistent with increased mechanical demands. Remarkably, these gaits did not differ in maximal velocities obtained: whether in Alligatoroidea or Crocodyloidea, trotting or bounding achieved similar velocities, revealing that the alligator lineage is capable of hitherto unappreciated extreme locomotor performance despite a lack of asymmetrical gait usage. Hence asymmetrical gaits have benefits other than velocity capacity that explain their prevalence in Crocodyloidea and absence in Alligatoroidea—and their broader evolution.

## Introduction

Extant Crocodylia have long been known to use almost all forms of walking and running locomotor modes (e.g., footfall patterns) present in quadrupedal mammals. These gaits include symmetrical (e.g., lateral/diagonal sequence walks; walking and running trots *vide*^1,2^) and asymmetrical (e.g., galloping, bounds and half-bounds) footfall patterns. Nevertheless, the diversity, scaling (body size correlations), and underlying mechanisms of this impressive locomotor repertoire are based only on a few studies of select species from the >23 extant members of Crocodylia. Symmetrical walking gaits have been fairly well studied for Crocodylia, mainly in alligatoroids such as *Alligator mississippiensis* (also *Caiman crocodilus*) [^3–20^ and see below]. There have been speculations or anatomical hints of divergent abilities in the “alligator lineage” (Alligatoroidea) vs. “crocodile lineage” (Crocodyloidea)^21–24^, but empirical evidence of any differences in locomotor abilities remains absent. Charig’s^25^ anecdote of sustained bipedal running in a crocodile (repeated in more recent studies; e.g.^26^) is highly specious, never having been reliably documented in that or any subsequent studies.

Bounding (synchronized left-right forelimb and hindlimb motions separated by an aerial phase) and galloping (slightly asynchronous left-right motions; essentially a slower version of bounding) gaits have been described for some *Crocodylus* species, especially *C. johnstoni*^27,28^, *C. porosus*^29^ and *C. niloticus*^30,31^. It is often misquoted in general media or natural history accounts that *C. johnstoni* is the only crocodylian known to gallop or bound; some^27^ wondered if its bounding ability was “unique” but it is clear that this capacity is more broadly distributed within Crocodylia. There is a brief report of putative asymmetrical gaits in juvenile *Gavialis*^5,32^, and anecdotes of these gaits in *C. palustris*, *C. novaeguineae* and *Osteolaemus tetraspis*^33^. It thus is poorly documented what species do use asymmetrical gaits — popular reviews of crocodylian biology often lament this gap in knowledge or recite old misinformation^34–36^.

In Alligatoroidea, studied velocities are almost exclusively slow, sustained walking <<2 ms^−1^ ^3–20^. When asymmetrical gaits are used by Crocodylia, they involve the fastest velocities that those species can attain on land, although few accurate measurements exist. Zug^29^ alleged bounding in *C. porosus* at up to 18 ms^−1^, which is faster than most land animals^37^; this was an error and ~1.8 ms^−1^ was intended^27^. Bornhauser and Ziswiler^31^ obtained similar velocities (0.4 to 2.0 ms^−1^) for a galloping *C. niloticus*. Velocities faster than 5 ms^−1^ have never been recorded for Crocodylia — up to 4.7 ms^−1^ maximal velocities were measured for *C. johnstoni*^27,28^, with similar estimates for *C. niloticus*^30^. Considering that bounding tends to be 2–4 times faster than trotting in *C. johnstoni*^27^, can species that do not use a gait faster than a relatively rapid, symmetrical trot (i.e. diagonal limbs in phase) only move 2–4 times slower than those that can use asymmetrical gaits?

The kinematics of asymmetrical gaits have been noted to be highly variable within and among Crocodylia, covering almost all possible footfall combinations^1,38^ and disparate correlations of velocity and kinematic parameters such as stride length or frequency^27–29^. Some of this variation appears size-related — Zug^29^ noted that only young *C. porosus* <2 m total length bound or gallop, and Webb and Gans^27^ claimed such gaits to occur “only in juveniles” of similar size. This apparent size bias has been speculated to be a biomechanical constraint^22,39^. Yet the dearth of empirical studies of asymmetrical gaits impairs broader understanding. Indeed, it remains uncertain what enables or constrains asymmetrical gait usage in Crocodylia. Complex interplay between vertebrae, osteoderms, connective tissue, skin and muscle^39–42^ as well as flexibility and stiffness of the intervertebral joints^43,44^ may be critical elements. This complexity is augmented by the fact that the axial column switches from its plesiomorphic function of lateral undulation to a derived dorsoventral undulatory motion in bounding and galloping Crocodylia^28,31^. The amount of lateral undulation was presumably reduced in more erect stem archosaurs and then increased back to near-ancestral levels in later Crocodylomorpha [e.g.^15,31^].

Despite intense study of locomotion in alligatoroids, at least at slow velocities, no members of this lineage have been clearly shown to truly bound or gallop^21^. Studies have also noted (sometimes subtle) differences in exercise physiology^45^, foot form and resulting tracks^46^, track-making kinematics^47^, pectoral girdle and humerus shape^48^, limb musculature and its allometry^22^ and limb or vertebral proportions^23,24^ that might relate to differences in locomotor function or even behavior among crocodylian lineages. Small *A. mississippiensis* “attempted to gallop”^11^ but a full stride was not achieved, and alligators “could not be induced to gallop”^9^. Numerous studies have proposed that asymmetrical gaits are ancestral for Crocodylia^21,27,39,49^, inherited from more terrestrial ancestors in Crocodylomorpha (or even Archosauria). Given that most prior data were based on studies of *C. johnstoni* and *C. porosus*, what can a broader sample of locomotor data from Crocodylia tell us about the evolution of asymmetrical gaits? Is there clear evidence for an ancestral capacity for bounding and galloping gaits in Crocodylia and do all lineages, at least at small body sizes, retain this ability?

Here we report our study of crocodylian locomotor dynamics that includes video documentation of gait usage in 15 species from a variety of body sizes within the range expected to move quickly (≤2 m total length; ≤50 kg body mass). First, we reconstruct the evolutionary history of asymmetrical gaits, which have been speculated to only be restricted to a few species. Using basic phylogenetic theory, we test the hypothesis (H1) that routine asymmetrical gait usage is homologous for all Crocodylia (i.e., present in Alligatoroidea and Crocodyloidea). Second, we quantify biomechanical constraints on gait usage in Crocodylia, especially the scaling of asymmetrical gait kinematics. We test the hypothesis (H2) that capacity for asymmetrical gaits declines with increasing body mass (i.e., negatively allometric scaling), for key kinematic parameters such as maximal velocity (absolute and size-normalized), minimal duty factor (relative ground contact time per stride; correlated with peak limb force^50^); and maximal relative stride frequency. Third, if we do find differences in gait usage (via H1 being falsified) between the lineages of Alligatoroidea and Crocodyloidea, we test the hypotheses that (H3) gait kinematics are different between asymmetrical and symmetrical gaits in the two clades; and (H4) asymmetrical gaits in Crocodyloidea are faster than symmetrical gaits in Alligatoroidea.

## Methods

We studied 42 individuals of 15 species of Crocodylia (body mass range 0.5 kg to 43 kg; Supplementary Dataset S1) at the St. Augustine Alligator Farm Zoological Park (Florida), outdoors during the daytime at mean summer temperatures ~30 °C (±2 °C approximately). An additional 14 individual Crocodylia (26 attempted trials; 12 species, including three not in the final dataset: *Paleosuchus trigonatus*, *Caiman jacare* and *Melanosuchus niger*) were studied but did not provide useful data and were excluded from the study; whereas 7 of our 42 individuals were measured twice in separate data collection sessions on different years (and here are treated as the same individual where relevant for statistical analyses). Crocodylians were caught from their enclosures, weighed and measured, and marked with white poster paint or infrared-reflective motion capture markers (1–2 cm diameter) around their joint centres of rotation. The animals were allowed to rest and recover from capture to minimize fatigue. Rest times between capture and between trials varied depending on keeper assessments of the condition of animals, and data collection sessions terminated when animals showed clear signs of fatigue or reluctance to locomote. Due to diverse constraints on available time for staff, experiments and animals, these rest times could not be standardized; nor could periodic cloacal temperature or blood lactate tests be performed.

For data collection, crocodylians were released at one end of a ~5 m long, 1–1.5 m wide runway on level ground, with its left central side facing a lateral view camera(s) and a second camera for dorsal view footage suspended above the central region. Animals were encouraged to move across the runway by simple release, auditory/visual cues, gentle prodding, and/or placement of refugia (bushes/water) at the end of the runway. The techniques used varied based on keepers’ advice customized to the individual animal and situation. The central runway flooring was either a force platform with top plate (force data not presented here), solid wooden board, or woodchips, depending on accessible space during the three years of data collection sessions (see below). Crocodylians were not harmed for the purposes of this study. The experimental protocol was reviewed and approved by the Royal Veterinary College’s Ethics and Welfare Committee; approval number URN 2012 1187 R. All experiments were performed in accordance with relevant guidelines and regulations.

The above methods were consistently used, but as we collected data during three different years (2002, 2004, 2005), the hardware and software used to collect and analyse the kinematic data varied. Video footage of trials was recorded in 2002 at 200 Hz (720 × 480 pixels), in 2004 at 60 Hz (720 × 480 pixels), and in 2005 at 50 Hz (720 × 576 pixels). All video data were then digitally captured (Ulead Visual Studio 9.0; Ulead Systems, Taipei, Taiwan) as video files trimmed down to individual trials for initial analysis in Virtual Dub software (http://www.virtualdub.org/), in which foot touchdown/liftoff timings (from video fields) were recorded for each visible limb for all complete strides (cycle of footfalls). We classified footfall patterns using the limb phases^1,2,28,38^ as a fraction of a stride between foot touchdown events; with the left hindlimb as the “0” reference. These limb phases corresponded to categories of footfall patterns (“gaits”) coded as trot (1), lateral sequence (2), diagonal sequence (3), rotary gallop (4), transverse gallop (5), half-bound (6), and bound (7); codes 1–3 were symmetrical gaits and 4–7 asymmetrical. We also calculated stance phase duration (time from touchdown to liftoff) and swing phase duration (time from liftoff to touchdown) for each limb, stride duration (mean stance + swing phase durations for all limbs), stride frequency (*SF*; inverse of stride duration; as Hz), and duty factor (*DF*; stance phase duration as a fraction of stride duration; as mean of all limbs) as kinematic parameters used in our statistical analyses.

In Matlab software (The MathWorks, Inc., Natick, MA), we digitized hip and shoulder markers from videos to calculate mean forward velocities (*u*) across a stride, using objects of known scale in the field of view to calibrate from pixels to meters of distance. In the 2005 data collection session, we also had four motion capture cameras (MCU 500; Qualisys AB, Göteborg, Sweden) arranged around the runway area; instead of a lateral view camera. These cameras were used to record (at 240 Hz) the 3D positions of the infrared markers around the body and limb joints of subjects, replacing the 50 Hz videos for velocity but not footfall analysis in that dataset. Only trials that were deemed to involve relatively straight-ahead, steady-state locomotion were used for kinematic analysis. Yet as crocodylians did not normally move quickly in a true steady state (e.g., >10% velocity change within a stride), we used a rough subjective criterion for “steady”, erring on the side of maximal inclusivity to favor natural – and near-maximal, where feasible – locomotor patterns (inevitably including variation that would introduce noise into our dataset). All valid occurrences of different gaits within that final dataset were recorded for characterizing which species used each locomotor mode.

To facilitate comparisons between Crocodylia of different sizes moving with comparable relative kinematics (i.e. more dynamically similar^51,52^), we normalized kinematic parameters. Relative stride frequency (*RSF*) was computed using *g* = 9.81 ms^−2^ and *h* = extended hindlimb length to tip of third digit (in m):

1 $$RSF=SF\cdot {(g\cdot {h}^{-1})}^{-0.5}$$

Dimensionless velocity ($\hat{u}$); or the square root of the Froude number; was:

2 $$\hat{u}=u\cdot {(g\cdot h)}^{-0.5}$$

Statistical analyses were conducted using SPSS Statistics software version 25 (IBM Corp., Armonk, NY). Phylogenetic statistics were not conducted as we had already split our sample into the lineages Crocodyloidea and Alligatoroidea and judged our sample inappropriate for available methods in this context. Linear models (LM) or linear mixed effects (LME) models (accounting for repeated measures from the same individuals using random effects) were used to assess hypotheses H2, H3 and H4. Residual variances were allowed to vary depending on the camera recording frequency (Hz) in both LM and LME models, because the higher frame rate and thus temporal precision of the 2002 (and 2004) datasets vs. 2005 might introduce non-systematic biases. Normality of the residuals was assessed visually and data were log-transformed where necessary. We analyzed three datasets: (1) all data for all velocities and gaits pooled (n = 42 individuals; 8 Alligatoroidea); (2) all “running” data obtained, where running was identified based on *DF* < 0.50 and an asymmetrical gait (n = 12 individuals, 36 strides); and (3) the single fastest stride (for $\hat{u}$ values ≥0.90) per individual (n = 22 Crocodyloidea; 5 Alligatoroidea), as follows.

H1 was tested qualitatively based on footfall patterns; the absence of asymmetrical gaits in Alligatoroidea (or Crocodyloidea) would falsify it. We tested H2 using datasets 2 and 3 (comparing patterns for our fastest vs. any running trials), with log body mass as the predictor and log *u*, log $\hat{u}$, *DF*, and *RSF* as the outcome variables. LME was used for analysis of all running trials (dataset 2), and LM was used for the analysis of each single fastest stride (dataset 3).

For testing H3, we used dataset 1 (all data), processed using LME as above; but comparing symmetrical gaits in Alligatoroidea with symmetrical and asymmetrical gaits in Crocodyloidea (i.e., three groups) to test for differences in adjusted means for log *u*, log $\hat{u}$, *DF*, and *RSF* between the three groups.

We tested H4 only with dataset 3 (fastest trial per individual with $\hat{u}$ ≥ 0.90), using LM, where only symmetrical gaits of Alligatoroidea were compared with asymmetrical gaits of Crocodyloidea.

All data including the videos of trials analyzed, and statistical code and analysis outputs, are available on Figshare [http://figshare.com/articles/Video_data_Crocodylian_locomotor_kinematics/11322035]. Additional kinematic changes with speed are described, using nonlinear regression analyses, in the Supplementary text S1, Supplementary Figs. S1–S3, and Supplementary Tables S1–S3.

## Results

**Figure 1:**
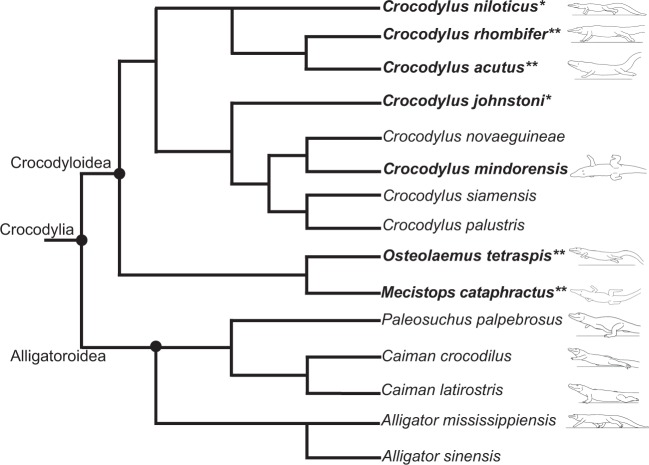
Distribution of asymmetrical gaits within Crocodylia. Examples of asymmetrical and symmetrical gaits (single frames from videos) from our analysis, mapped onto a phylogeny of Crocodylia (composite from^67–71^); for testing our H1. Taxa in bold font are known to use asymmetrical gaits. *Indicates taxa with recorded asymmetrical gaits in prior studies and this one; **Indicates taxa with new discoveries of asymmetrical gaits in this study. Line drawings on the right side (by Scott Hartman) are outlines from screen captures from experimental videos of the fastest strides of representative individuals from dataset 3, taken from visible hindfoot-off timings and emphasizing symmetrical gaits for Alligatoroidea vs. bounding asymmetrical gaits for Crocodyloidea. Overhead views of *Crocodylus mindorensis* and *Mecistops cataphractus* were reversed so that all are facing left. Not to scale.

We obtained 184 useable trials from slow to near-maximal velocities (0.15–4.4 ms^−1^). 42 trials involved asymmetrical gaits, from 17 of our 42 individual subjects (Supplementary Dataset S2). Supplementary Movies S1–S10 illustrate the diverse range of rapid symmetrical and asymmetrical gait performance that we recorded across Crocodylia. We found that Crocodyloidea and Alligatoroidea used different gaits — the former adopted a wide range of asymmetrical gaits (normally a bound at their fastest velocity^28^), whereas the latter only employed symmetrical gaits (and normally a trot at their fastest velocities). No Alligatoroidea ever galloped or bounded; thus our Hypothesis 1 (H1) was not supported (Fig. 1).

We recorded the first documentation of asymmetrical gaits in the critically endangered Philippine crocodile *Crocodylus mindorensis* — all four of the juveniles studied were remarkably adept at the full range of crocodylian gaits (e.g., Supplementary Movie S6). Likewise, we obtained data for asymmetrical gaits (1 subject, 2 trials; rotary gallop at 2.36 ms^−1^ and bounding at 2.98 ms^−1^) in the critically endangered African slender-snouted crocodile *Mecistops cataphractus*. Additionally, we recorded the only published dataset for asymmetrical gait usage in *C. rhombifer* (Supplementary Movie S8), *C. acutus* and (in quantitative detail; cf. Cott, 1961; Bornhauser and Ziswiler, 1983) *C. niloticus* (Supplementary Movie S7); plus one instance of a very rapid symmetrical gait (3.1 ms^−1^ trotting) in *C. siamensis*. Previous evidence for terrestrial locomotor behavior in these taxa was anecdotal at best. We also confirmed purported bounding and galloping ability in *Osteolaemus tetraspis* (Whitaker and Andrews, 1988), providing the only quantitative data on asymmetrical gaits in this unusual dwarf crocodile, such as bounding at up to 2.98 ms^−1^ (Supplementary Movie S9). Our data for the more well-studied *C. johnstoni* contributed new quantitative information on gaits of adult individuals, augmenting a detailed study of small juveniles^28^, although our adults did not reach the most rapid velocities of those animals or the field study subjects^27^.

Our study’s measurements of high speed locomotion in alligatoroids are novel. For example, the fastest published, reliably recorded velocities are 0.62 ms^−1^ for fast trotting in juvenile *A. mississippiensis*, with duty factors (*DF*) ~0.70^10,14^. In contrast, the maximal velocities and minimal *DF* we obtained were >3 ms^−1^ and <0.50 *DF* for alligatoroids, with our fastest young *A. mississippiensis* trotting at 2.0 ms^−1^ and 0.50 *DF* (Supplementary Movie S1).

**Figure 2:**
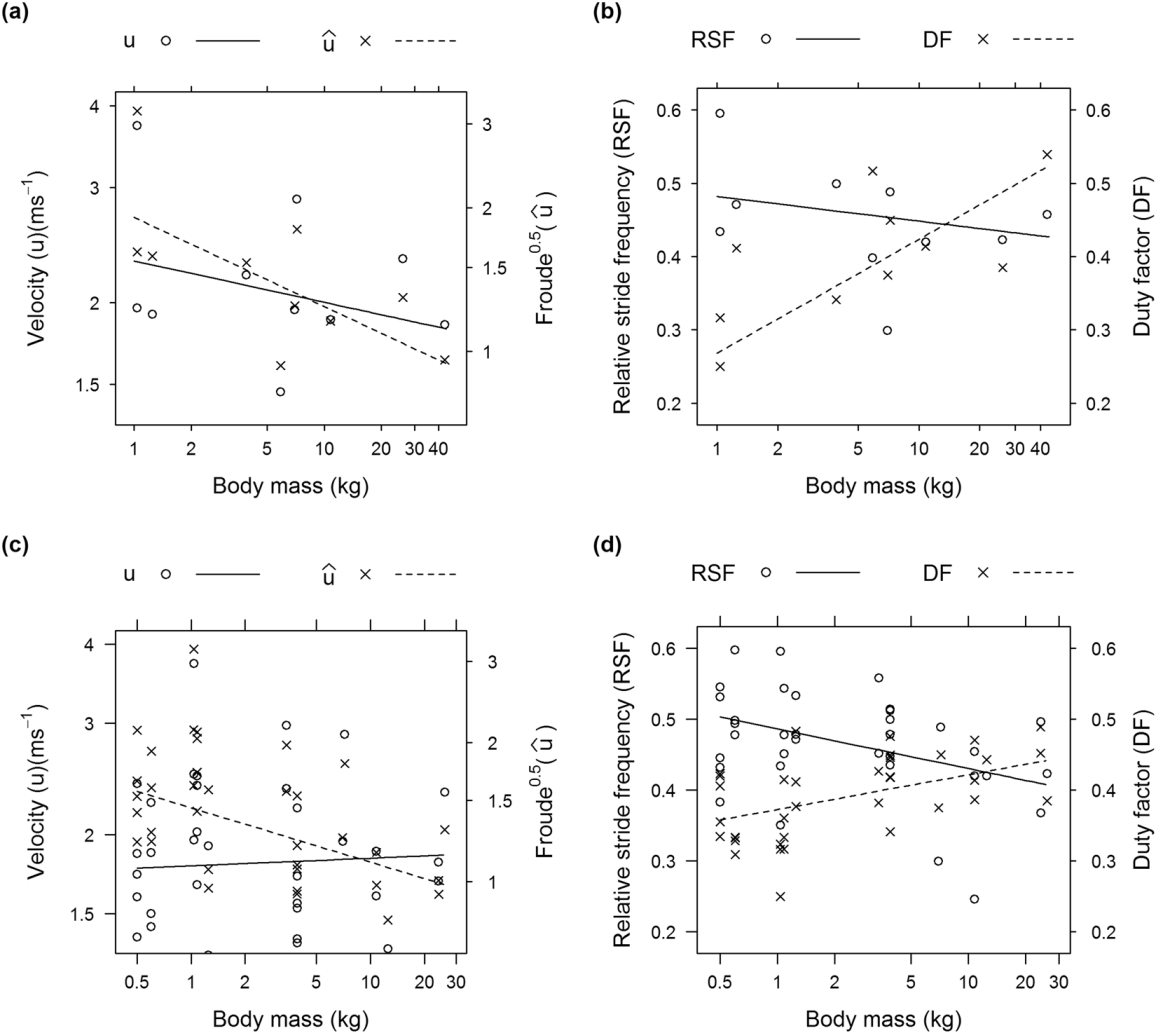
Bivariate plots from analyses using linear mixed effects models (**a,b**) and linear models (**c,d**), depicting the relationships of kinematic y-variables with body mass, based on only Crocodyloidea and asymmetrical gait data. (**a,b**): dataset 3 (fastest running stride per individual); (**c,d**): dataset 2; all “running” strides (*DF* < 0.50), accounting for repeated measures per individual. See Table 1 for adjusted means and standard errors of the coefficients.

Body mass did not have a significant correlation with maximal velocity (*u*; in ms^−1^) although our subjects’ size range, despite spanning two orders of magnitude (0.5–43.2 kg), was restricted to smaller, presumably faster individuals (~2 m total length or less) under 50 kg body mass, far from the >500 kg mass that some of our study species can reach (Fig. 2). However, we found body mass effects on size-normalized kinematics across our subjects that indicated a decline in dimensionless locomotor performance — at the maximal velocities measured (dataset 3), heavier Crocodylia had slower relative velocities ($\hat{u}$) and greater *DF* (p = 0.026, 0.013). Thus smaller Crocodylia were more athletic, with greater capacity for asymmetrical gaits as per our H2. As dataset 3 was a limited sample size (n = 10 individual Crocodyloidea using asymmetrical gaits at their maximal *u*), we checked if these results were still upheld with our dataset for all Crocodyloidea using fast asymmetrical gaits (dataset 2; n = 12 individuals, 36 trials), which produced congruent correlations; additionally emphasizing that *RSF* decreased weakly with body mass (p = 0.014; Table 1). These findings are robust support for our Hypothesis 2 (H2) that the capacity for asymmetrical gaits declines with increasing body mass (i.e., negatively allometric scaling) within Crocodylia; particularly Crocodyloidea.

**Table 1:** Results from tests of H2 and H3, from a linear mixed effects model analysis (see Methods), focusing on four kinematic parameters: log velocity (*u*), log Froude^0.5^ ($\hat{u}$), duty factor (*DF*) and relative stride frequency (*RSF*).

Test + data	log(*u*)	log($\hat{{\boldsymbol{u}}}$)	*DF*	*RSF*
H2: dataset 3	−0.06 ± 0.03	**−0.19 ± 0.03**	**0.07 ± 0.01**	−0.01 ± 0.02
maximal *u*	(p = 0.180)	**(p = 0.026)**	**(p = 0.013)**	(p = 0.417)
H2: dataset 2	0.01 ± 0.05	**−0.12 ± 0.04**	**0.02 ± 0.01**	**−0.02 ± 0.01**
running (*DF* < 0.50)	(p = 0.806)	**(p = 0.044)**	**(p = 0.029)**	**(p = 0.014)**
H3: Alli-symm	0.05 ± 0.21^ab^	−0.41 ± 0.22^ab^	**0.57 ± 0.03**^**a**^	**0.30 ± 0.03**^**a**^
H3: Crocs-symm	**−0.05 ± 0.11**^**a**^	**−0.48 ± 0.12**^**a**^	**0.56 ± 0.02**^**a**^	**0.30 ± 0.02**^**a**^
H3: Crocs-asymm	**0.46 ± 0.14**^**b**^	**0.04 ± 0.14**^**b**^	**0.46 ± 0.02**^**b**^	**0.41 ± 0.02**^**b**^
H3: Overall p-value	**<0.0001**	**<0.0001**	**<0.0001**	**<0.0001**

Residual variances were allowed to vary depending on the recording frequency (Hz). Bold font emphasizes where p < 0.05. For H2, relationships with log(body mass) are based only on Crocodyloidea and asymmetrical gait data. Adjusted regression coefficients (i.e. slope of the regression line) ± standard errors are shown. Results from dataset 3 are compared with those from dataset 2, in which the model accounted for repeated measures from the same subjects. For H3, we present a comparison of three groups (“alli-symm” = Alligatoroidea symmetrical gaits; “crocs-symm” = Crocodyloidea symmetrical gaits; “crocs-asymm” = Crocodyloidea asymmetrical gaits), using dataset 1 (all valid strides), focusing on adjusted means ± standard errors. Individual number was used as a random effect in the analysis. There was no statistical difference between groups sharing the same letters (superscript a or b).

**Figure 3:**
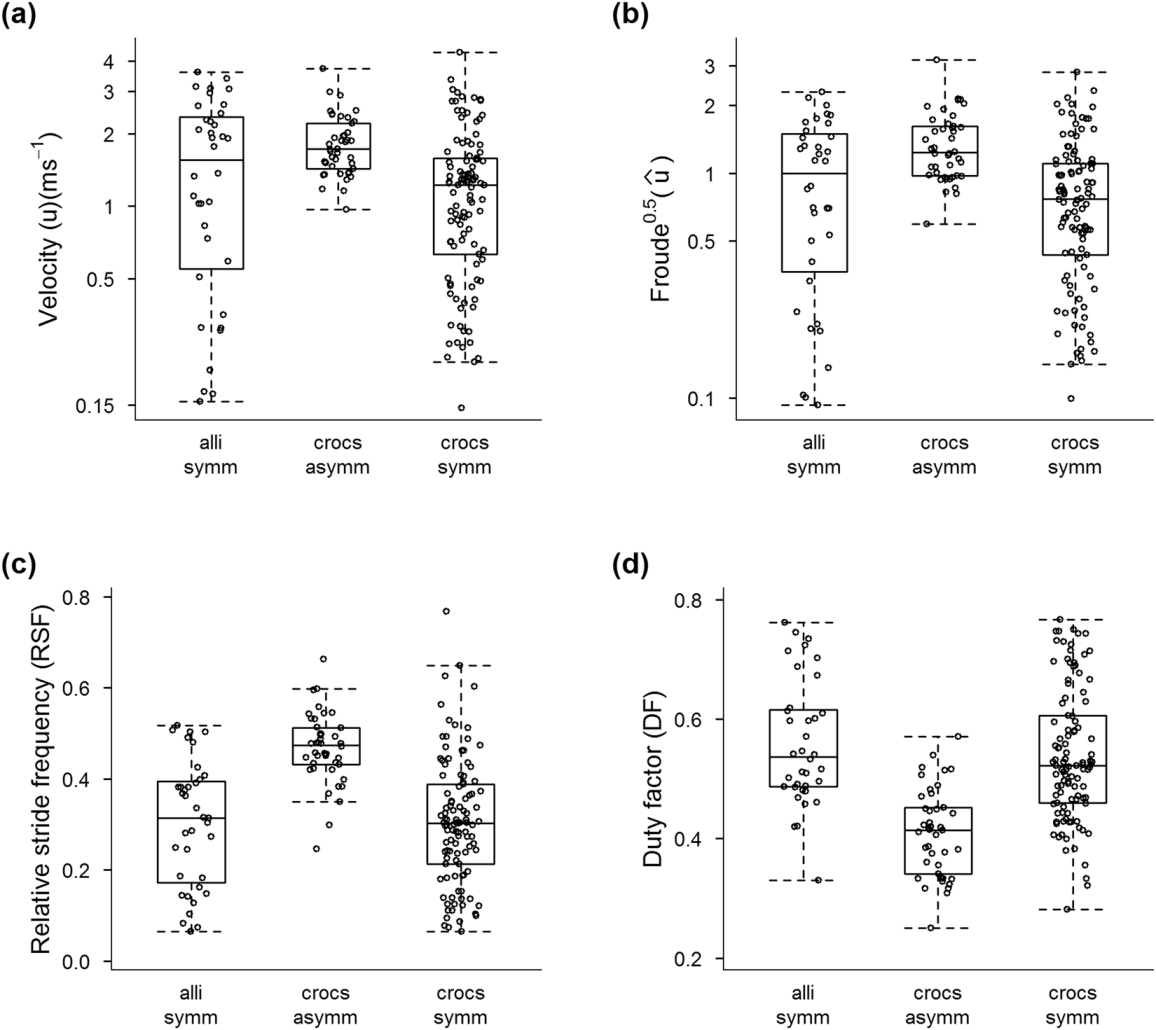
Box-and-whisker plots comparing four kinematic parameters (**a–d**) for three categories of locomotor data from Crocodylia (“alli symm” = Alligatoroidea symmetrical gaits; “crocs asymm” = Crocodyloidea asymmetrical gaits; “crocs symm” = Crocodyloidea symmetrical gaits), based on dataset 1 (all gait data). See Table 1 for adjusted means and standard errors from the linear mixed effects modeling analyses.

Considering that our H1 was falsified, we inspected what kinematic parameters differentiated Crocodyloidea and Alligatoroidea for all velocities and gaits (dataset 1; symmetrical and asymmetrical gaits). Our analysis (Fig. 3) supported Hypothesis 3, that symmetrical vs. asymmetrical gait kinematics differ between the two major clades of Crocodylia (p < 0.0001; Table 1). Velocities in ms^−1^ were only different within Crocodyloidea: asymmetrical gaits tended to be faster than symmetrical gaits; but across all trials and subjects Crocodyloidea was no faster than Alligatoroidea. This same pattern was reproduced when we inspected $\hat{u}$. Yet asymmetrical gaits had greater *RSF* and smaller *DF* than symmetrical gaits (e.g., trotting), both within Crocodyloidea and for Crocodyloidea vs. Alligatoroidea; a trend that was also observable within individuals (Supplementary Dataset S2). Symmetrical gaits, in contrast, did not differ between the two clades of Crocodylia. Other aspects of basic locomotor kinematics were indistinguishable between the two major lineages of Crocodylia, although there was high variability (Fig. 3; Supplementary Figs. S1–S3).

**Figure 4:**
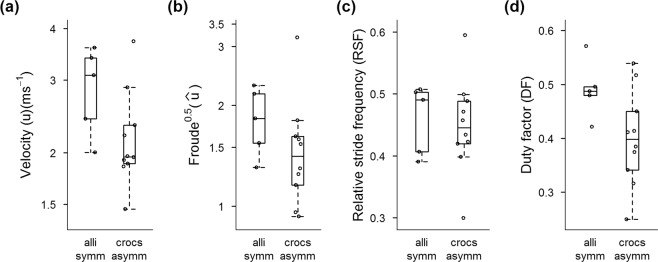
Box-and-whisker plots comparing four kinematic parameters (**a–d**) for two clades of Crocodylia (“alli symm” = Alligatoroidea symmetrical gaits; “crocs asymm” = Crocodyloidea asymmetrical gaits) based on dataset 3 (fastest running stride per individual). See Table 2 for adjusted means and standard errors from the linear modeling analyses.

Our Hypothesis 4 posited that asymmetrical gaits are faster than symmetrical gaits (from dataset 3). The maximal velocities (*u*) and $\hat{u}$ in each individual studied for Alligatoroidea vs. Crocodyloidea (Fig. 4) showed unexpected differences: in particular, Crocodyloidea was ~30–50% slower than Alligatoroidea (p = 0.030, 0.031; Table 2). However, given the small sample sizes and high variation we are wary of accepting that inference, leaving our H4 open to interpretation but certainly disfavouring slower absolute or relative velocities for Alligatoroidea. Notably, we obtained a maximal *u* of 3.7 and 4.4 ms^−1^ for our fastest individuals from these two clades (Supplementary Dataset S2); respectively *Caiman crocodilus*, trotting (Supplementary Movie S3); and *Crocodylus acutus*, using a diagonal sequence running gait (Supplementary Movie S2). We also did not find any prominent differences between *DF* or *RSF* for Alligatoroidea vs. Crocodyloidea, although *DF* was weakly smaller (by 0.07 or ~14% vs. Alligatoroidea) in the latter clade (p = 0.075).

**Table 2:** Results from tests of H4, from a linear mixed effects model analysis (see Methods), focusing on four kinematic parameters: log velocity (*u*), log Froude^0.5^ ($\hat{u}$), duty factor (*DF*) and relative stride frequency (*RSF*) from dataset 3 (fastest running stride per individual); comparing the adjusted means ± standard errors for two groups (“alli-symm” = Alligatoroidea symmetrical gaits; “crocs-asymm” = Crocodyloidea asymmetrical gaits); with p values (bold font for < 0.05). Residual variances were allowed to vary depending on the recording frequency (Hz).

Data	log(*u*)	log($\hat{{\boldsymbol{u}}}$)	*DF*	*RSF*
Alli-symm	**1.02 ± 0.10**	**0.60 ± 0.10**	0.49 ± 0.03	0.47 ± 0.03
Crocs-asymm	**0.72 ± 0.07**	**0.28 ± 0.08**	0.42 ± 0.02	0.45 ± 0.02
H4: Overall p-value	**0.030**	**0.031**	0.075	0.437

Furthermore, 25 individuals of 13 species (87 trials in our broader dataset 2; including symmetrical gait data) achieved relatively rapid running gaits with aerial phases (mean *DF* < 0.5), with 5 and 4 of these individuals and species (~25% of our sample) being from Alligatoroidea. We often found that, within individual crocodiles using both symmetrical and asymmetrical running gaits, both gait categories could reach similar velocities (e.g., 17 individuals used asymmetrical gaits but it was their fastest gait in only 10 of them; Supplementary Dataset S2). For example, one 0.5 kg *C. mindorensis* used a diagonal sequence symmetrical gait at 2.44 ms^−1^ and a transverse gallop at 2.41 ms^−1^; its two fastest trials; in addition to a lateral sequence symmetrical gait at 1.99 ms^−1^ and a half-bound at 1.87 ms^−1^. Crocodyloidea that bounded, nonetheless, usually used it as their fastest gait.

## Discussion

First, we have shown that only members of Crocodyloidea in our sample used asymmetrical gaits, falsifying our H1. Second, our H2 was supported, that capacity for asymmetrical gait usage declines with increasing body mass (from 0.5 to 43 kg; two orders of magnitude in scaling) in crocodiles. Third, we demonstrated that there are no clear differences between the symmetrical gait kinematics of Alligatoroidea and Crocodyloidea, but that asymmetrical gaits in crocodiles involve greater relative stride frequencies and smaller duty factors than symmetrical gaits in either lineage (supporting H3). Finally, our H4 was falsified — Alligatoroidea and Crocodyloidea can reach similar speeds even though only the latter uses asymmetrical gaits to achieve them. We now explore the implications of each hypothesis test.

Our data establish that small representatives of Crocodyloidea, whether immature members of large-bodied species or adult members of dwarf species (e.g., *Osteolaemus*), can bound and gallop, and this capacity seems to be ancestral at least for this lineage. Most studies have concluded that asymmetrical gaits were ancestral for Crocodylia, based on the functional morphology of fossil Crocodylomorpha [^39–41,49^ but see^44^]. We concur that the morphofunctional evidence renders this the most plausible hypothesis. If that scenario is correct, then ancestral Alligatoroidea lost this ability (or do not express it). Conversely, an alternative hypothesis that the common ancestor of Crocodylia lacked the capacity for asymmetrical gaits and Crocodyloidea uniquely evolved it still deserves examination; a possibility that is almost never acknowledged. The reported presence of asymmetrical gaits in young *Gavialis*^5,32^ is important because if that taxon lies outside of Crocodyloidea + Alligatoroidea (Fig. 1) then this would bolster the inference that asymmetrical gaits are ancestral for Crocodylia. The locomotion of its purported sister taxon in Crocodyloidea, *Tomistoma*, remains unstudied to our knowledge. Indeed, asymmetrical gaits in *Gavialis* are not documented with concrete photo or video imagery and hence deserve empirical examination.

Why alligatoroids do not bound or gallop remains uncertain. Some candidate explanations such as muscle leverage or exercise physiology are not known to be appreciably different for these clades but have not been deeply investigated. Habitats frequented by these lineages of Crocodylia differ, with some Alligatoroidea ranging into more temperate climes. Here, we studied all of our Crocodylia at about the same ambient “field” temperature, which they had been acclimatized to in captivity, so this variable was removed in our experimental design but body temperature was not explicitly controlled.

We have also made discoveries and confirmed anecdotes about previously obscure behaviors of Crocodylia: featuring the first documented evidence of asymmetrical gaits in *Crocodylus mindorensis*, *C. rhombifer*, *C. acutus, Mecistops cataphractus* and *Osteolaemus tetraspis* (Fig. 1; Supplementary Movies S6, S8, S9). These records are important given the critically endangered status of the first three taxa. Anecdotes of asymmetrical gait usage in *C. palustris* and *C. novaeguineae*^33^ are now plausible in light of the prevalence of these gaits in other Crocodyloidea. We were unable to obtain more than symmetrical gaits from these two taxa; respectively only 2 and 3 valid trials each. Together, our new observations of asymmetrical gaits and our broader dataset on locomotor kinematics spanning the clade Crocodylia considerably expand our knowledge of their behaviours and natural history. Importantly, this combined evidence strongly refutes the popular notion that only a few crocodiles (mainly *C. johnstoni*; also *C. porosus*) use asymmetrical gaits.

Webb and Gans^27^ noted that smaller *Crocodylus johnstoni* used greater stride frequencies than larger individuals, but these freshwater crocodiles were capable of increasing maximal velocities across a snout-vent length range of ~20–85 cm, consistent with positive allometry of absolute athletic performance (albeit within less than one order of body size range). As *DF* in our analysis would have a strong inverse correlation with ground reaction forces incurred by the limbs^50^, our observation of increased *DF* in larger Crocodyloidea (across two orders of magnitude of body mass range) is consistent with data from muscle architecture, demonstrating negative allometry of the relative capacity to generate muscular forces in proportion to body weight^22^. This pattern of reduced relative athletic capacity is strongly reinforced by the negative allometry of $\hat{u}$ and *RSF*—larger crocodiles moved more slowly relative to their size using fewer strides per unit time. At some body mass beyond the range of our subjects’, true running gaits with *DF* < 0.50 should become impossible.

Interestingly, symmetrical gait kinematics did not differ between Crocodyloidea and Alligatoroidea in our sample, but asymmetrical gaits had smaller *DF* and greater *RSF*; hence involving more extreme kinematics at a given speed. This result (from H3, focusing on dataset 1) was somewhat reflected at maximal speeds (H4; dataset 3; Table 2 for *DF*) although differences were marginal. Overall, our data for Crocodyloidea correspond well with the few detailed records for bounding and galloping kinematics. In particular, for *Crocodylus johnstoni*, Renous *et al*.^28^ obtained minimal *DF* ~0.2 and maximal $\hat{u}$ >1.0, with *SF* > 2.5 Hz, and their individuals used asymmetrical gaits between 0.4–4 ms^−1^, only bounding past 2.0 ms^−1^, and generally maintaining forelimb greater than hindlimb *DF* values; matching general kinematic patterns in this study (e.g., Supplementary Text S1; Supplementary Figs. S1–S3; Supplementary Tables S1–S3).

In addition to their importance for basic understanding of maximal performance in Crocodylia, these findings have implications for bone safety factors (ratios of failure stress to peak *in vivo* stress). Past experimental assessment of bone safety factors^10^ has made important contributions towards understanding crocodylian functional design but may have underestimated safety factors at maximal performance and, thus, deserves some re-evaluation. Perhaps the limb bones of Crocodylia are not as “overbuilt” as previously inferred^10^ because those calculations were based on locomotor speeds and gaits that impose lower stresses on the bones, thereby reflecting local maximum bone stresses of that given speed/gait rather than the absolute maximum (‘peak’) stresses for a species/individual. The extreme minimal *DF* values presented here, close to 0.40 (vs. 0.70 in prior studies), imply that peak limb forces (and thus tissue stresses) should be ~1.75 times greater than prior measurements^10^, which would lead to proportionately reduced safety factors (e.g., from mean values respectively for the femur and tibia of 6.7 and 2.7 to 3.8 and 1.5). However, Blob and Biewener^10^ also calculated “worst case” values of 3.2 and 1.3, which our data suggest reducing to 1.8 and 0.74 (with the caveat that these are purely theoretical values). The revised estimates suggested here, while making the assumption that limb angular kinematics (and thus moments) do not change with speed or gait, nonetheless fall near the ~2–4 range of safety factors estimated for birds and mammals^53^.

Thus more investigation of how peak “field” (non-laboratory) performance might impact conclusions drawn from safety factor analysis, or other characterizations of locomotion, is needed for Crocodylia. The grossly similar morphology of limb bones across Crocodylia^23^ and the prevalence of asymmetrical gaits in Crocodyloidea, involving some faster speeds and smaller duty factors (hence even greater loads^50^), are further cause for caution. Prior studies of Alligatoroidea recorded maximal speeds of 0.62 ms^−1^ or less and *DF* ≥ 0.66^3–20^. These are undeniably valuable data on high walks/slow running trots at close to preferred or moderate speeds, but this study shows even the fastest prior data are far from maximal speeds or minimal duty factors.

We acknowledge challenging limitations to this study. It was difficult to motivate many individuals, hence 14 from our initial sample were excluded, and the majority of these (nine total) were from Alligatoroidea. Indeed, we noticed that members of the alligator lineage seemed (qualitatively) more likely to sit and hiss, struggle, or fight rather than run away from stimuli or release from captivity, compared with Crocodyloidea. We speculate that this is a behavioral tendency that may partly underlie the divergent locomotor abilities within Crocodylia. Even so, our main sample was disproportionately represented by Crocodyloidea (34 vs. 8 Alligatoroidea individuals), which partly was by design. Intensive prior studies of the alligator lineage have failed to identify any asymmetrical gaits in this clade, so we focused on collecting data from under-sampled species (e.g., not *Alligator mississippiensis*) and from individuals expected by keepers to be highly active—these constraints limited our sample of Alligatoroidea. Regardless, we found hitherto unreported extreme locomotor performance in this clade, albeit only using symmetrical gaits. Our conclusion that Alligatoroidea does not employ asymmetrical gaits could be reinvestigated with a broader sample, but our study and existing literature on Crocodyloidea and Alligatoroidea strongly point toward a divergence in their gait usage.

Performance of individual Crocodylia varied tremendously, and surely was influenced by complex factors including not only motivation but also fatigue, body temperature, personnel, equipment, environment (e.g. substrate stiffness) and more. Our statistical models took into account the main factors we could identify as likely confounding agents, and our experimental design attempted to maximally control these factors within the constraints of the setting, staff, and animals. As an additional check, we re-ran the LME analyses for H2 and H4 (repeated measures) including the effects of stride and trial numbers within an individual as covariates in the models, to test if later strides or trials within our datasets had slower speeds. We found no such effect – indeed, where any effect was found it was a very slight increase of speed in later trials. Hence we conclude that animals did not suffer clear fatigue across trials.

The surprising result that maximal velocities did not differ between Alligatoroidea and Crocodyloidea (H4; Fig. 4, Table 2), or were sometimes even faster in Alligatoroidea, reveals that the key benefit of asymmetrical gaits in Crocodylia is not maximal speed capacity. Thus a vexing mystery is why Crocodyloidea bound or gallop, given that they tend to choose to do so at faster speeds, and why this capacity originated, if more deeply embedded within Crocodylomorpha. Allen *et al*.^22^ inferred that longer limb muscle fascicles correlate with asymmetrical gaits in Crocodylia (also see^54^). Webb and Gans^27^ postulated that asymmetrical gaits in *Crocodylus johnstoni* are useful for crossing rough terrain. A study of mouse gaits suggested that bounding might have benefits for stability against perturbations^55^, consistent with the latter notion and paralleling suggestions of stability/maneuverability tradeoffs in bounding and galloping *C. johnstoni*^28^. Reilly *et al*.^56^ also proposed benefits for velocity and energetics in bounding toads; aspects of metabolism related to fatigue or endurance remain unexplored candidate explanations for asymmetrical gaits in Crocodylia.

Asymmetrical galloping and bounding gaits are recognized to have evolved multiple times in sarcopterygian vertebrates, including lungfish^57^, toads^56^, turtles^54^, and mammals [e.g.^1,58^] — and at least one spider and one insect use analogous mechanisms^59,60^. Thus these locomotor mechanisms are far from being restricted to cursorial mammals as some analyses imply [e.g.^61–63^]. Notably, as in prior studies^27–31^, all of our Crocodyloidea using asymmetrical gaits had a single extended period (e.g., Fig. 1) during their aerial phase (“suspension”), not a single gathered (“collected”) suspension or alternating gathered/extended suspensions as typify various mammals^61,62^. The reasons for this singular axial undulatory pattern remain obscure. The aforementioned cases of convergent evolution provide potential to understand the truly fundamental principles of these gait mechanisms vs. which patterns (e.g., axial undulatory motions) are divergent. Such understanding could test if stability or other benefits, such as circumventing breathing constraints^64^, broadly underlie the evolution of asymmetrical gaits, and could be useful in crocodylian-inspired robotic design [e.g.^65,66^].

## Supplementary information

Movie S1

Movie S2

Movie S3

Movie S4

Movie S5

Movie S6

Movie S7

Movie S8

Movie S9

Movie S10

Supplementary Information

Dataset S1

Dataset S2
