# Supplementary material for: Divergent evolution of terrestrial locomotor abilities in extant Crocodylia
Source: Sci Rep. 2019 Dec 17;9:19302. doi: 10.1038/s41598-019-55768-6 (PMC6917812; doi:10.1038/s41598-019-55768-6)
Supplement: Supplementary file 11 — Supplementary Information [file 41598_2019_55768_MOESM11_ESM.docx]

Supplementary Information for

Divergent evolution of terrestrial locomotor abilities in extant Crocodylia

John R. Hutchinson, Dean Felkler, Kati Houston, Yu-Mei Chang, John Brueggen, David Kledzik and Kent A. Vliet

Corresponding author: John R. Hutchinson

Email: [jhutchinson@rvc.ac.uk](mailto:jhutchinson@rvc.ac.uk)

**This file includes:**

Supplementary text S1

Figs. S1 to S3

Tables S1 to S3

Supplementary file captions

Supplementary Text

**Explanation of supplementary figures.** Figs. S1–S3 depict basic kinematic parameters for all 184 experimental gait data points to explore changes with dimensionless speed. All plots were made with GraphPad Prism version 8.0.0 for Windows (GraphPad Software, San Diego, CA; [www.graphpad.com](http://www.graphpad.com)) using nonlinear exponential curve fits with default assumptions (one-phase decay). The intention was purely exploratory, to describe overall speed-related (and size-normalized) patterns, not to test any hypotheses regarding differences in kinematics between clades, species, individuals, strides, gaits, or other categories; hence all data were analyzed together.

The nonlinear exponential models follow:

Y = (Y_0_ – P) * exp (-K * X) + P (equation S1)

Where Y_0_ is the value of Y at x = 0 (“intercept”), P is the plateau (y approaches as x ~ infinite), and K is the rate constant (“slope”).

Fig. S1 shows a general decrease from (non-normalized) stance and swing times of 2 and 0.7 sec respectively at the slowest dimensionless speeds to 0.05 and 0.15 sec at the fastest dimensionless speeds; i.e. declining by 40x and 2.1x. Fig. S2 presents how duty factor declines about three-fold from ~0.76 to 0.25 across the same speed range, whereas the difference between forelimb and hindlimb duty factors (*DF*) shows a trend for forelimb *DF* to become closer to hindlimb *DF* or even greater than it at the fastest dimensionless speeds, although there is wide variation in this “*DF* diff”. Fig. S3 demonstrates a fairly linear increase of relative stride length with dimensionless speed, from a minimum of ~1.25 to maximum of 5.36 (i.e., by 4.29x); whereas relative stride frequency’s relationship is more strongly curvilinear, increasing from 0.06 to ~0.7 (i.e., by 11.7x). Thus, overall, Crocodylia tend to increase speed mainly via decreasing stance time (and thus duty factor) and increasing stride frequency, although decreases in swing time and increases of stride length also contribute to speed increases (e.g., see Renous et al., 2002 cited in main text).


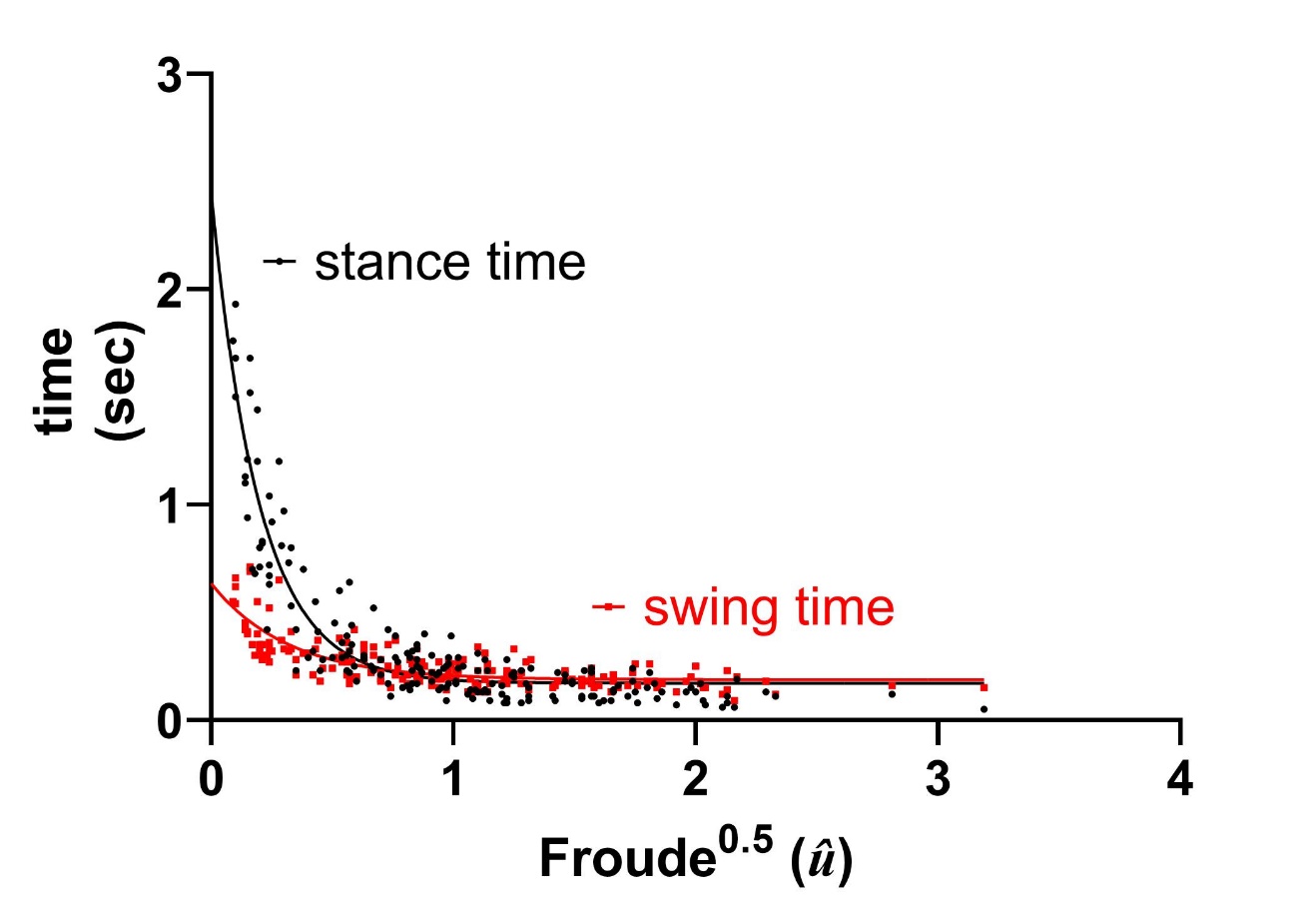
**Fig. S1.** Changes in stance and swing time (average for all 4 limbs) for all 184 trials of Crocodylia gait kinematics, plotted against dimensionless speed ($\hat{\boldsymbol{u}}$). See Supplementary text for methods, and Table S1 for summary statistics.

Fig. S2. Changes in duty factor (*DF*; average for all 4 limbs), and difference in *DF* of forelimbs vs. hindlimbs, for all 184 trials of Crocodylia gait kinematics, plotted against dimensionless speed ($\hat{\boldsymbol{u}}$). See Supplementary text for methods, and Table S2 for summary statistics.


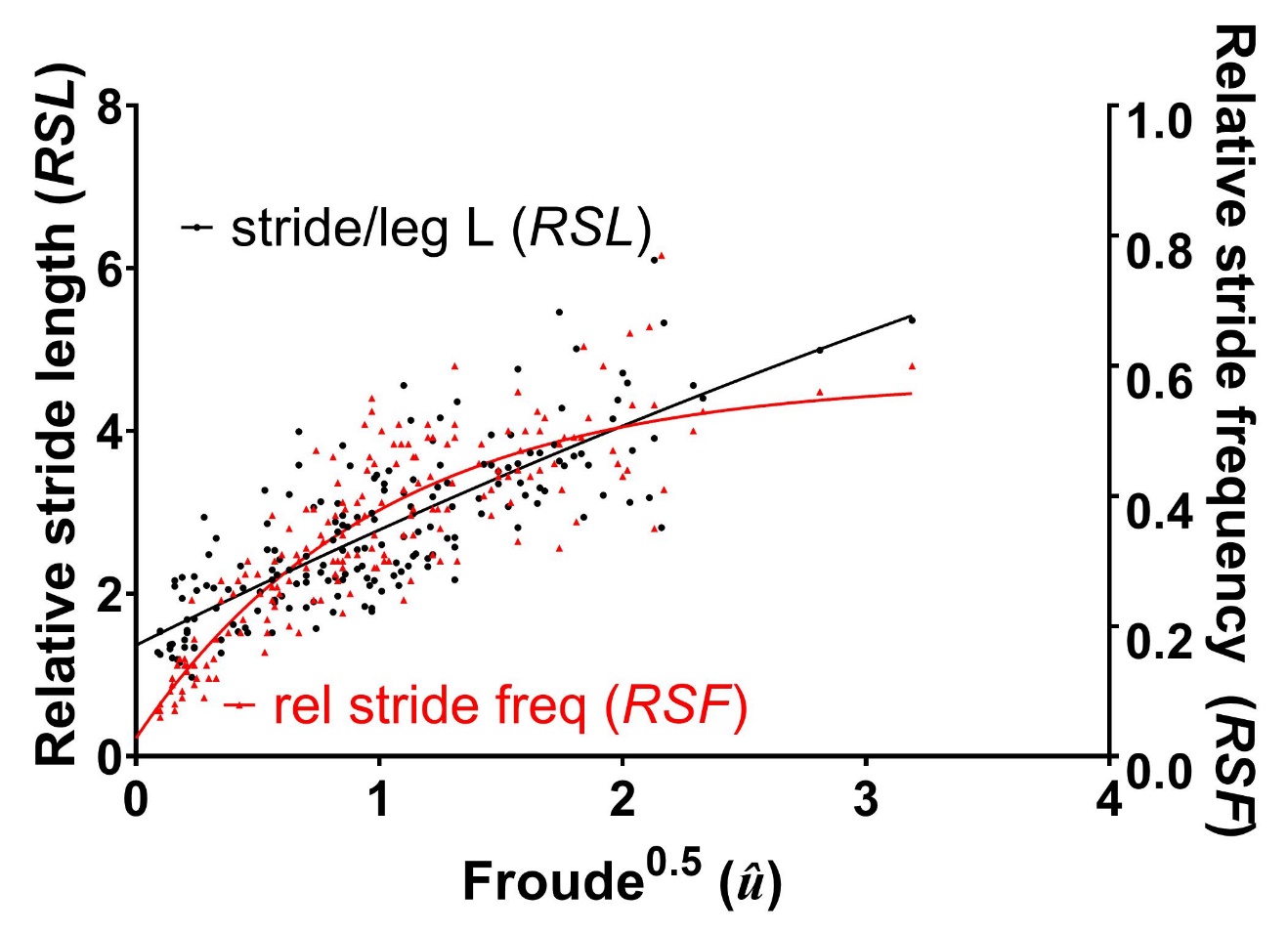


Fig. S3. Changes in relative stride length (*RSL*) and relative stride frequency (*RSF*) for all 184 trials of Crocodylia gait kinematics, plotted against dimensionless speed ($\hat{\boldsymbol{u}}$). See Supplementary text for methods and Table S3 for summary statistics.

Table S1. Summary statistics for Fig. S1.

| Curve fit | stance t | swing t |
| --- | --- | --- |
| Y_0_ | 2.44 | 0.633 |
| P | 0.171 | 0.187 |
| K | 5.04 | 3.14 |
|  |  |  |
| 95% CIs |  |  |
| Y_0_ | 2.14, 2.81 | 0.549, 0.757 |
| P | 0.141, 0.199 | 0.164, 0.206 |
| K | 4.26, 5.95 | 2.21, 4.51 |
|  |  |  |
| Goodness of fit |  |  |
| DoF | 181 | 181 |
| R^2^ | 0.855 | 0.620 |
| N | 184 | 184 |

Table S2. Summary statistics for Fig. S2.

| Curve fit | DF | DF diff |
| --- | --- | --- |
| Y_0_ | 0.802 | -0.0330 |
| P | 0.388 | -0.00159 |
| K | 1.55 | 1.30 |
|  |  |  |
| 95% CIs |  |  |
| Y_0_ | 0.756, 0.854 | -∞ to -0.00550 |
| P | 0.344, 0.419 | -0.0199 to +∞ |
| K | 1.12, 2.06 | ??? to +∞ |
|  |  |  |
| Goodness of fit |  |  |
| DoF | 181 | 181 |
| R^2^ | 0.688 | 0.0108 |
| N | 184 | 184 |

Table S3. Summary statistics for Fig. S3.

| Curve fit | RSL | RSF |
| --- | --- | --- |
| Y_0_ | 0.0275 | 1.36 |
| P | 0.580 | 15.7 |
| K | 1.01 | 0.104 |
|  |  |  |
| 95% CIs |  |  |
| Y_0_ | -0.0177, 0.0692 | 1.10, 1.59 |
| P | 0.523, 0.669 | 6.21 to +∞ |
| K | 0.713, 1.34 | ??? to 0.403 |
|  |  |  |
| Goodness of fit |  |  |
| DoF | 181 | 181 |
| R^2^ | 0.762 | 0.651 |
| N | 184 | 184 |

Supplementary File Captions

Supplementary Movies

Movie S1. Video (left side view) for trial “AMA_6” (see Dataset S2), showing *Alligator mississippiensis* trotting at 2.00 ms^-1^. Video details: .AVI format, 1.407 MB, 720x480 pixels, 30 Hz (but fields = 200 Hz), H.264 compression.

Movie S2. Video (top view) for trial “CA1_4c” (see Dataset S2), showing *Crocodylus acutus* using a diagonal sequence running gait at 4.35 ms^-1^. Video details: .AVI format, 0.877 MB, 768x576 pixels, 25 Hz, H.264 compression.

Movie S3. Video (left side view) for trial “CACRB_1A” (see Dataset S2), showing *Caiman crocodilus* trotting at 3.59 ms^-1^. Video details: .AVI format, 0.455 MB, 720x540 pixels, 30 Hz, H.264 compression.

Movie S4. Video (top view) for trial “CC1_5b” (see Dataset S2), showing *Mecistops* (“*Crocodylus*”) *cataphractus* trotting at 3.05 ms^-1^. Video details: .AVI format, 1.560 MB, 768x576 pixels, 25 Hz, H.264 compression.

Movie S5. Video (top view) for trial “CJ1_2” (see Dataset S2), showing *Crocodylus johnstoni* using a lateral sequence running gait at 2.72 ms^-1^. Video details: .AVI format, 0.915 MB, 768x576 pixels, 25 Hz, H.264 compression.

Movie S6. Video (top view) for trial “CM1_3b” (see Dataset S2), showing *Crocodylus mindorensis* bounding at 3.73 ms^-1^. Video details: .AVI format, 0.771 MB, 768x576 pixels, 25 Hz, H.264 compression.

Movie S7. Video (left side view) for trial “CNA_19” (see Dataset S2), showing *Crocodylus niloticus* bounding at 2.20 ms^-1^. Video details: .AVI format, 1.305 MB, 720x480 pixels, 30 Hz (but fields = 200 Hz), H.264 compression.

Movie S8. Video (left side view) for trial “CRB_6” (see Dataset S2), showing *Crocodylus rhombifer* bounding at 2.00 ms^-1^. Video details: .AVI format, 1.85 MB, 720x480 pixels, 30 Hz (but fields = 200 Hz), H.264 compression.

Movie S9. Video (left side view) for trial “OTI_2C” (see Dataset S2), showing *Osteolaemus tetraspis* bounding at 2.88 ms^-1^. Video details: .AVI format, 0.482 MB, 720x540 pixels, 30 Hz, H.264 compression.

Movie S10. Video (left side view) for trial “PPD_1B” (see Dataset S2), showing *Paleosuchus palpebrosus* using a lateral sequence running gait at 3.08 ms^-1^. Video details: .AVI format, 0.517 MB, 720x540 pixels, 30 Hz, H.264 compression.

Supplementary Datasets

Dataset S1 (separate file)

Subject information. “Crocodile codes” worksheet contains acronyms for each genus/species used in other worksheets. “Crocodyloidea” and “Alligatoroidea” worksheets contain metadata for each subject in those two clades. Metadata comprises year of measurements, crocodile acronym (individual code), coded species number, coded year number (1 = 2002; 2 = 2004; 3 = 2005) for experimental data collection, individual number within species, individual number total, leg length in meters, and body mass in kg. Identical data are compiled in Dataset S2 with corresponding experimental data but this condensed dataset is provided to facilitate access to subject metadata.

Dataset S2 (separate file)

Experimental kinematic datasets. “Crocodile codes” worksheet contains acronyms for each genus/species used in other worksheets. “Datasets 1-3” contain all kinematic data used in this study (including supplementary analyses), corresponding to the dataset numbers described in the text; 1 = all data; 2 = all running data; 3 = only maximal speed data (see Methods for details). Metadata comprises year of measurements, crocodile acronym (individual code), video number (code used in Figshare repository data files), coded species number, coded year number (1 = 2002; 2 = 2004; 3 = 2005) for experimental data collection, individual number within species, individual number total, trial number for individual, stride number within trial, gait number (see Methods), gait classification (0 = symmetrical; 1 = asymmetrical), camera recording frequency, subject leg length in meters, subject body mass in kg, velocity in ms^-1^, *û*, *u* in leg lengths per second, stance time, swing time, *DF*, hindlimb and forelimb mean *DF*, forelimb – hindlimb *DF* (“*DF* diff”), right hind- (RH) and right fore- (RF) and left fore- (LF) limb phases as fraction of stride vs. LH contact (not used in analyses but reported here for comparisons with gait numbers), stride frequency and *RSF*, and stride length and *RSL*.
